# Risk of infections in patients with gout: a population-based cohort study

**DOI:** 10.1038/s41598-017-01588-5

**Published:** 2017-05-03

**Authors:** B. Spaetgens, F. de Vries, J. H. M. Driessen, H. G. Leufkens, P. C. Souverein, A. Boonen, J. W. M. van der Meer, L. A. B. Joosten

**Affiliations:** 1 Division of Pharmacoepidemiology and Clinical Pharmacology, Utrecht Institute for Pharmaceutical Sciences, Utrecht University, Utrecht, The Netherlands; 2 Department of Internal medicine and Rheumatology, Maastricht University Medical Centre+, Maastricht, The Netherlands; 3 Care and Public Health Research Institute (CAPHRI), Maastricht University, Maastricht, The Netherlands; 4 Department of Clinical Pharmacy and Toxicology, Maastricht University Medical Centre+, Maastricht, The Netherlands; 5 Department of Internal Medicine, Radboud Institute of Molecular Life Sciences (RIMLS), Radboud University Medical Centre, Nijmegen, The Netherlands

## Abstract

To investigate the risk of various types of infections (pneumonia and urinary tract infection (UTI)), and infection-related mortality in patients with gout compared with population-based controls. A retrospective cohort study was conducted using data from the UK Clinical Practice Research Datalink (CPRD). All patients with a first diagnosis of gout and aged >40 years between January 1987-July 2014, were included and matched with up to two controls. Time-varying Cox proportional hazards models were used to estimate the risk of infections and mortality. 131,565 patients and 252,763 controls (mean age: 64 years, 74% males, mean follow-up of 6.7 years) were included in the full cohort. After full statistical adjustment, the risk of pneumonia was increased (adj. HR 1.27, 95% CI 1.18 to 1.36), while the risk of UTI (adj. HR 0.99, 95% CI 0.97 to 1.01) was similar in patients compared to controls. No differences between patients and controls were observed for infection-related mortality due to pneumonia (adj. HR 1.03, 95% CI 0.93 to 1.14) or UTI (adj. HR 1.16, 95% CI 0.98 to 1.37). In conclusion, patients with gout did not have decreased risks of pneumonia, UTI or infection-related mortality compared to population-based controls.

## Introduction

Gout is worldwide the most common type of inflammatory arthritis with estimates of prevalence ranging from 2.5% in Europe to 3.9% in the United States^1–3^. It is a chronic disease with different disease manifestations varying from acute self-limiting attacks to chronic tophaceous gout^4^. Gout and the accompanying hyperuricaemia have also been associated with a large number of comorbidities, such as cardiovascular disease, hypertension, diabetes and chronic kidney disease, although it remains unclear whether there is a causal relation with these comorbidities^5, 6^.

The pathogenesis of gout is well understood, with a major role for monosodium urate (MSU) crysals which are formed during a state of hyperuricaemia^7^. In the joints these MSU crystals elicit an inflammatory response by activation of the NLRP3 inflammasome, which eventually leads to production of the pro-inflammatory cytokine interleukin (IL)-1bèta^8–10^. Also, in patients with tophaceous gout, it has been shown that pro-inflammatory cytokines such als IL-1bèta are expressed in the tophus, suggesting a state of chronic inflammation induced and/or stimulated by MSU crystals^11^. Isolated peripheral blood mononuclear cells (PBMCs) of patients with gout produce more IL-1bèta when stimulated with MSU, when compared to blood cells of healthy controls^12^. Further, MSU seems to enhance the function of these PBMCs by epigenetic mechanisms resulting in increased cytokine production (‘Trained Immunity’)^13^. Recent evidence further revealed that the production of IL-1bèta and IL-6 were higher in patients with gout, and serum concentrations correlated with serum uric acid (sUA)^14^.

As such, the net result of aforementioned pathways suggest a pro-inflammatory state in patients with hyperuricaemia and/or gout^14^.

The presence of a pro-inflammatory state related to hyperuricaemia has led to the hypothesis that patients with gout may have an enhanced resistance to infections, because previous research indicated that IL-1bèta augments the quality of host defence against bacteria and viruses^15^. However, the clinical correlate of this concept has never been explored. Since gout is classically treated with colchicine for acute flares, and with allopurinol for long-term uric acid lowering treatment (ULT), it is important to consider a potential effect of these drugs on the relation between gout and infections. As such, we hypothesized that (I) patients with gout acquire fewer community-acquired infections (e.g., pneumonia, urinary tract infection (UTI)) and encounter a lower infection-related mortality rate, (II) treatment with colchicine enhances infections, because of its immunosuppressive effects, and (III) treatment with allopurinol neutralizes the protective role of high sUA levels of infections.

In view of the above, the objective of this study was to investigate the risk of various types of infections (pneumonia and UTI), and infection-related mortality in patients with gout compared with population-based controls without gout.

## Patients and Methods

### Design and data source

A retrospective cohort study was conducted using data from the British Clinical Practice Research Datalink (CPRD) GOLD (January 1987–July 2014). CPRD is formerly known as the General Practice Research Database (GPRD) and contains the computerized medical records of approximately 13 million patients under care of general practitioners in the United Kingdom (UK) who are representative for 6.9% of the total UK population. Practices contribute to CPRD, only when their data quality is up to research standards. Since 1987, data recorded in the CPRD include demographic information, prescription details, lifestyle parameters, clinical events, preventive care provided and specialist referrals. CPRD has been extensively validated^16^, and used previously to study gout^17^, and infections including pneumonia^18^. About 75% of all practices in England (58% of all UK CPRD practices) were linked to data of the Office of National Statistics (ONS) (January 1998–January 2012)^19^. The ONS provided data for the causes of death and the exact date as recorded on death certificates by a medical doctor. Linkage of death certificates to CPRD has a high level of validity^20^.

## Ethical approval

CPRD has been granted Multiple Research Ethics Committee approval (05/MRE04/87) to undertake purely observational studies with external data linkages, including ONS mortality data. The present study is based on anonymised and unidentifiable CPRD data and is also approved by the Independent Scientific Advisory Committee (ISAC) of the Medicine and Healthcare products Regulatory Agency (protocol number 14_123R), which is primarily responsible for reviewing the study protocols. In the case of this observational research no further ethical approval deemed necessary by ISAC.

### Study population

All patients with a first diagnosis of gout during the period of valid data collection (from 1 January 1987 to 30 June 2014) and aged above 40 years during the period of valid CPRD data collection were included. Each patient with gout was matched by year of birth, sex, and practice to up to two patients without a diagnosis of gout using incidence density sampling. The date of the first recorded diagnosis of gout defined the index date and controls were assigned the same index date as their matched patient with gout. Patients or controls with a history of exposure to colchicine and ULT (allopurinol, febuxostat and/or uricosuric drugs) before index date were excluded.

When the outcome infection-related mortality was evaluated, data were restricted to the participants with linkage to ONS data. For this analysis, the period of valid data collection was restricted from 1 January 1998 to 31 December 2011.

### Study outcome(s) and covariates

The primary outcomes of interest were a first event of pneumonia (specified by read codes) or UTI (specified by read codes or prescriptions for nitrofurantoin or trimethoprim, since in the UK nitrofurantoin is exclusively, and trimethoprim in 95% of all cases prescribed for the treatment of UTI). The secondary outcome of interest was infection-related mortality (death related to pneumonia [International Classification of Diseases, Tenth Revision (ICD-10) codes: J10.0-J18.9] or UTI [ICD-10 codes: N39.0]).

Potential confounders assessed at baseline included sex, body mass index (BMI), smoking status and alcohol use. Age and an estimated glomerular filtration rate (eGFR) were included as time-dependent variables at the start of each (30-day) time interval. When pneumonia or infection-related mortality due to pneumonia was the outcome, potential confounders included a history of pneumonia (more than 3 months ago), a history of sinusitis, influenza infection, stroke, lung cancer, chronic obstructive pulmonary disease (COPD), acute bronchitis, asthma, diabetes, dementia, epilepsy/seizures, dysphagia, HIV/AIDS and the use of the following medications within the previous 6 months: antipsychotics, acid suppressants, bronchodilators/inhaled corticosteroids, anticonvulsants, immunosuppressants or systemic glucocorticoids and within 1 year prior: an influenza vaccination, and within 5 year prior: a pneumococcal vaccination. When UTI or infection-related mortality due to UTI were the outcome, potential confounders included a history of diabetes, malignancies excluding non-melanoma skin cancer, HIV/AIDS, recent use (within the previous 3 months) of urinary catheters, and the use of immunosuppressants or systemic glucocorticoids in the 6 months before. All confounders but age were treated as categorical variables.

### Statistical Analysis

Analyses were conducted using time-varying Cox proportional hazard models to estimate the risk of each outcome with exposure to gout. Patients were followed from the index date up to the end of data collection, the outcome of interest, the date of transfer out of the practice area or death, whichever came first. Follow-up was stratified into periods of 30 days. Potential confounders were included in the final model if they independently changed the bèta-coefficient of the univariate analysis by ≥5%, or when consensus (within the research team) about inclusion was reached, supported by scientific evidence. Analyses were repeated after stratification for treatment (current (<31 days), recent (31–91 days), past (>91 days) users) with allopurinol or colchicine. Data management and statistical analyses were conducted using SAS 9.3 (PHREG procedure).

## Results

**Figure 1:**
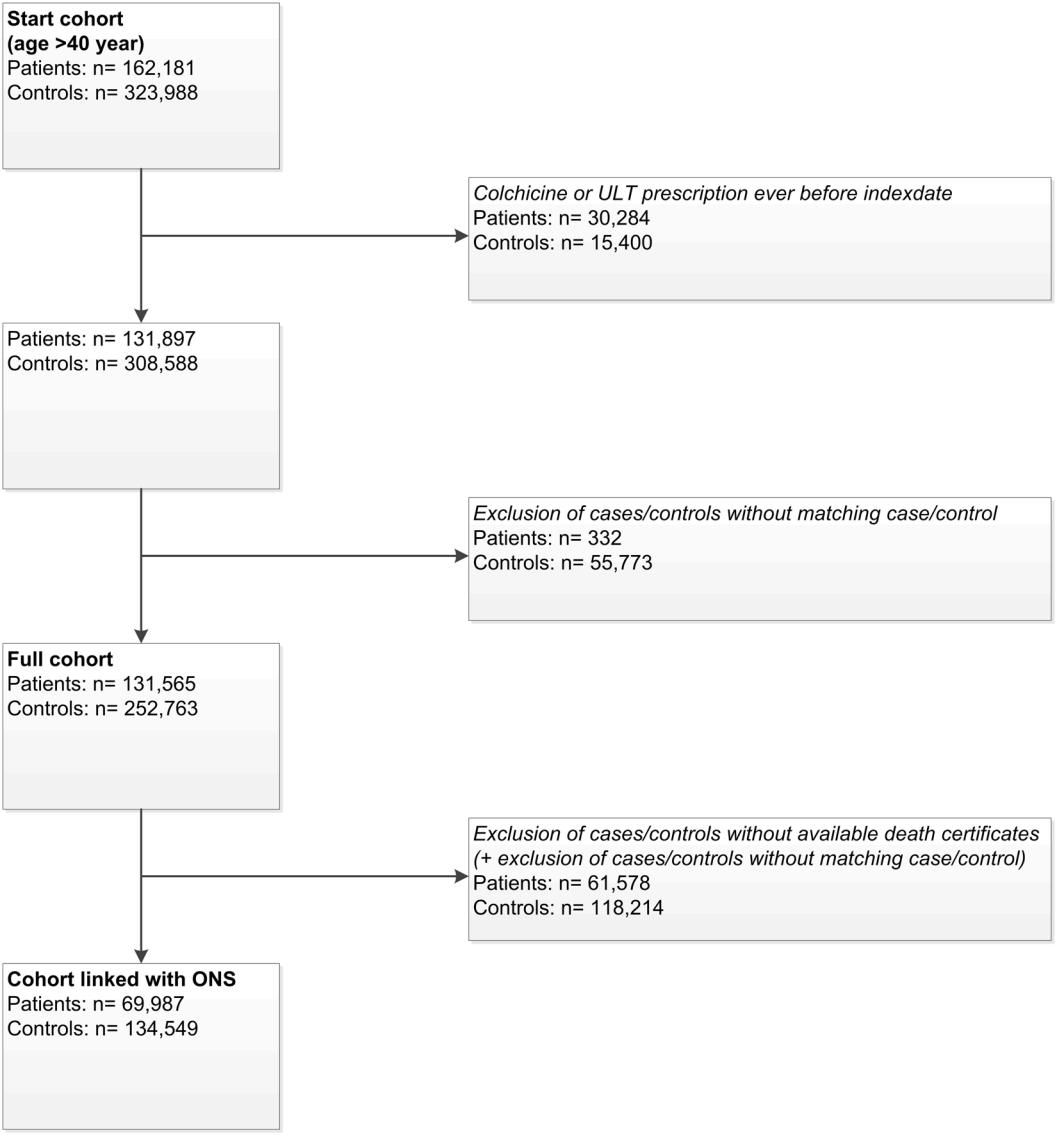
Flowchart of the study subjects.

In total, 162,181 patients with gout and 323,988 controls aged 40+ years were identified. Next, 30,284 gout patients and 15,400 controls with a prescription of colchicine or ULT before index date were excluded, and a remaining 56,105 subjects who were no longer matched. As a result, 131,565 patients with gout were included along with 252,763 controls without gout (mean age: 64 years, 74% males) with a mean follow-up of 6.7 years for both patients and controls in our full cohort. When infection-related mortality was the outcome 69,987 patients and 134,549 controls for whom death-certificate data were available for analyses after linkage to the ONS (Fig. 1) were included.

Baseline characteristics of patients with gout and controls in both cohorts are shown in Table 1. Patients with gout had, compared to controls, a higher BMI, more often an eGFR below 60 mL/min/1.73, consumed more often alcohol, suffered more frequently of asthma and/or COPD, diabetes, ischemic heart disease and atrial fibrillation. Furthermore, patients with gout were more likely users of systemic glucocorticoids or bronchodilators. No differences were observed for a history of malignancies, HIV or use of urinary catheters or immunosuppressant drugs.

**Table 1:** Baseline characteristics of patients with gout and controls, separately for the full sample and the sample that could be linked to death certificate data from the Office of National Statistics (ONS)^a^.

Characteristics	Full Cohort		Cohort linked with ONS	
	Gout patients n = 131,565	Controls n = 252,763	Gout patients n = 69,987	Controls n = 134,549
Mean follow-up time, years (SD)	6.7 (5.1)	6.7 (5.1)	7.3 (4.9)	7.2 (5.0)
Number of men	97,179 (73.9)	186,021 (73.6)	52,024 (74.3)	99,612 (74.0)
Age				
Mean age at index date, years (SD)	64 (13.5)	64 (13.5)	64 (13.5)	64 (13.5)
40–49 years	23,304 (17.7)	46,032 (18.2)	12,474 (17.8)	24,607 (18.3)
50–59 years	27,968 (21.3)	54,730 (21.7)	14,967 (21.4)	29,380 (21.8)
60–69 years	31,262 (23.8)	59,875 (23.7)	16,614 (23.7)	31,896 (23.7)
70 + years	49,031 (37.3)	92,126 (36.4)	25,932 (37.1)	48,666 (36.2)
BMI				
Mean BMI at index date, kg/m^2^ (SD)	29 (5.2)	27 (4.8)	29 (5.2)	27 (4.8)
<20.0 kg/m^2^	1,756 (1.3)	7,461 (3.0)	954 (1.4)	4,030 (3.0)
20.0–24.9 kg/m^2^	21,146 (16.1)	63,823 (25.3)	11,624 (16.6)	35,430 (26.3)
25.0–29.9 kg/m^2^	46,675 (35.5)	80,677 (31.9)	25,449 (36.4)	43,679 (32.5)
30.0–34.9 kg/m^2^	25,308 (19.2)	30,167 (11.9)	13,510 (19.3)	15,747 (11.7)
≥35.0 kg/m^2^	12,107 (9.2)	10,909 (4.3)	6,143 (8.8)	5,609 (4.2)
Missing	24,573 (18.7)	59,726 (23.6)	12,307 (17.6)	30,054 (22.3)
Smoking status				
Never	62,964 (47.9)	118,635 (46.9)	33,274 (47.5)	63,441 (47.2)
Current	26,517 (20.2)	60,059 (23.8)	14,715 (21.0)	32,704 (24.3)
Ex	38,941 (29.6)	60,101 (23.8)	20,961 (29.9)	32,625 (24.2)
Missing	3,143 (2.4)	13,968 (5.5)	1,037 (1.5)	5,779 (4.3)
Alcohol use				
No	18,772 (14.3)	40,138 (15.9)	9,473 (13.5)	20,179 (15.0)
Yes	101,993 (77.5)	178,735 (70.7)	55,652 (79.5)	98,483 (73.2)
Missing	10,800 (8.2)	33,890 (13.4)	4,862 (6.9)	15,887 (11.8)
eGFR most recent within previous year				
eGFR, ml/min/1.73 m^2^ (SD)	73 (22.7)	81 (22.0)	73 (22.7)	81 (22.0)
>90 ml/min/1.73 m^2^	14,769 (11.2)	34,509 (13.7)	7,456 (10.7)	17,412 (12.9)
60–89 ml/min/1.73 m^2^	38,052 (28.9)	63,485 (25.1)	21,267 (30.4)	35,780 (26.6)
30–59 ml/min/1.73 m^2^	19,251 (14.6)	16,428 (6.5)	10,771 (15.4)	9,306 (6.9)
15–29 ml/min/1.73 m^2^	1,609 (1.2)	878 (0.3)	889 (1.3)	499 (0.4)
<15 ml/min/1.73 m^2^	183 (0.1)	186 (0.1)	106 (0.2)	106 (0.1)
Missing	57,701 (43.9)	137,277 (54.3)	29,498 (42.1)	71,446 (53.1)
History of comorbidities				
Pneumonia	3,280 (2.5)	4,801 (1.9)	1,735 (2.5)	2,653 (2.0)
UTI	13,010 (9.9)	21,979 (8.7)	6,828 (9.8)	11,599 (8.6)
HIV	70 (0.1)	156 (0.1)	18 (0.0)	59 (0.0)
Lung cancer	207 (0.2)	460 (0.2)	103 (0.1)	224 (0.2)
Malignancies^b^	14,363 (10.9)	25,684 (10.2)	7,729 (11.0)	13,706 (10.2)
Asthma/COPD	18,829 (14.3)	29,940 (11.8)	9,903 (14.1)	15,852 (11.8)
Sinusitis	14,754 (11.2)	26,043 (10.3)	7,920 (11.3)	14,181 (10.5)
Diabetes	11,151 (8.5)	16,952 (6.7)	5,872 (8.4)	8,731 (6.5)
Dementia	985 (0.7)	3,071 (1.2)	448 (0.6)	1,472 (1.1)
Epilepsy	2,143 (1.6)	4,523 (1.8)	1,091 (1.6)	2,333 (1.7)
Dysphagia	2,371 (1.8)	4,169 (1.6)	1,248 (1.8)	2,259 (1.7)
Ischaemic heart disease	19,492 (14.8)	24,177 (9.6)	10,132 (14.5)	12,651 (9.4)
Atrial fibrillation/flutter	11,544 (8.8)	10,972 (4.3)	6,016 (8.6)	5,848 (4.3)
Stroke	7,681 (5.8)	11,457 (4.5)	3,888 (5.6)	5,908 (4.4)
Drug use within six months before				
Bronchodilators	14,423 (11.0)	21,495 (8.5)	7,426 (10.6)	11,128 (8.3)
Inhaled corticosteroids	7,052 (5.4)	10,902 (4.3)	3,811 (5.4)	5,989 (4.5)
Antipsychotics	1,252 (1.0)	3,602 (1.4)	630 (0.9)	1,878 (1.4)
Anticonvulsants	2,785 (2.1)	5,671 (2.2)	1,367 (2.0)	2,894 (2.2)
Systemic corticosteroids	5,538 (4.2)	7,915 (3.1)	2,908 (4.2)	4,177 (3.1)
Immunosuppressants	835 (0.6)	1,549 (0.6)	465 (0.7)	798 (0.6)
Influenza vaccination (1 year before)	48,379 (36.8)	80,038 (31.7)	28,345 (40.5)	48,188 (35.8)
Pneumococcal vaccination (5 years before)	23,174 (17.6)	38,350 (15.2)	12,846 (18.4)	21,805 (16.2)

Abbreviations: SD, standard deviation; ONS, the Office of National Statistics; BMI, Body mass index; eGFR, estimated Glomerular Filtration Rate; HIV, Human Immunodeficiency Virus; COPD, Chronic Obstructive Pulmonary Disease.

a) Data represent the number (%) of patients, unless stated otherwise.

b) Excluding non-melanoma skin cancer.

Table 2 shows that gout was associated with a 34% increased risk of pneumonia, after adjustment for age and sex (HR 1.34, 95% CI 1.25 to 1.43). Full statistical adjustment led to a marginal reduction in the risk estimate (adjusted (adj.) HR 1.27, 95% CI 1.18 to 1.36). Further stratification by use of gout medication showed that there was no increased risk of pneumonia with current colchicine use (adj. HR 0.88, 95% CI 0.54 to 1.44), while recent and past use was associated with a doubled pneumonia risk. Current exposure to allopurinol was associated with an increased risk of pneumonia (adj. HR 1.41, 95% CI 1.23 to 1.61), which was similar compared to the risk of non-allopurinol use. Discontinuation of allopurinol barely altered the risk of pneumonia.

**Table 2:** Risk of pneumonia in patients with gout compared to matched controls, stratified by use of colchicine and allopurinol.

	No. of pneumonia events (n = 3,586)	Age/sex adjusted HR (95% CI)	Adjusted HR^a^ (95% CI)
No Gout	2,048	Reference	Reference
Gout	1,538	1.34 (1.25 to 1.43)	1.27 (1.18 to 1.36)
By colchicine exposure			
Never use	1,159	1.24 (1.15 to 1.33)	1.22 (1.13 to 1.31)
Current use (<31 days)	16	1.16 (0.71 to 1.90)	0.88 (0.54 to 1.44)
Recent use (31–91 days)	32	2.09 (1.48 to 2.97)	1.60 (1.13 to 2.27)
Past use (>91 days ago)	331	1.80 (1.60 to 2.02)	1.49 (1.32 to 1.68)
By allopurinol exposure			
Never use	954	1.22 (1.13 to 1.32)	1.18 (1.10 to 1.28)
Current use (<31 days)	262	1.65 (1.45 to 1.87)	1.41 (1.23 to 1.61)
Recent use (31–91 days)	119	1.76 (1.46 to 2.11)	1.58 (1.31 to 1.91)
Past use (>91 days ago)	203	1.43 (1.24 to 1.65)	1.39 (1.21 to 1.61)

Abbreviations: HR: indicates hazards ratio; CI, confidence interval; PY, person years.

(a) Adjusted for age, sex, body mass index, smoking status, alcohol use, the most recently recorded estimated glomerular filtration rate in the past year, a history of dementia, dysphagia, and the use of bronchodilators, inhalation corticosteroids, systemic corticosteroids, non-insulin anti-diabetic drugs, insulin within 6 months before, the use of influenza vaccinations 1 year before and the use of pneumococcal vaccinations 5 years before.

Table 3 shows that gout was associated with a 14% increased risk of UTI, after adjustment for age and sex (HR 1.14, 95% CI 1.12 to 1.17). After full statistical adjustment this increased risk disappeared (adj. HR 0.99, 95% CI 0.97 to 1.01). The following confounders were responsible for this shift: sex, a history of chronic kidney disease, cancer, stroke, recent use of a urinary catheter and the use of systemic corticosteroids or anti-diabetics 6 months prior. Further stratification by the use of medication showed an increased risk of UTI with current and recent colchicine use (adj. HR 1.42, 95% CI 1.24 to 1.64 and adj. HR 1.29, 95% CI 1.12 to 1.49), whereas past colchicine use was not associated with an increased risk (adj. HR 1.00, 95% CI 0.95 to 1.06) compared to controls. Current and recent use of allopurinol was associated with a similar risk of UTI compared to controls (adj. HR 1.05, 95% CI 0.99 to 1.12 and adj. HR 1.04, 95% CI 0.94 to 1.14, respectively). However, past use of allopurinol was associated with a 27% reduced risk of UTI (adj. HR 0.73, 95% CI 0.67 to 0.80).

**Table 3:** Risk of urinary tract infection (UTI) in patients with gout compared to matched controls, stratified by use of colchicine and allopurinol.

	No. of UTI Events (n = 35,807)	Age/sex adjusted HR (95% CI)	Adjusted HR^a^ (95% CI)
No Gout	22,719	Reference	Reference
Gout	13,088	1.14 (1.12 to 1.17)	0.99 (0.97 to 1.01)
By colchicine exposure			
Never use	10,596	1.11 (1.08 to 1.14)	0.97 (0.94 to 1.01)
Current use (<31 days)	225	1.83 (1.62 to 2.07)	1.42 (1.24 to 1.64)
Recent use (31–91 days)	235	1.71 (1.52 to 1.93)	1.29 (1.12 to 1.49)
Past use (>91 days ago)	2,032	1.27 (1.22 to 1.33)	1.00 (0.95 to 1.06)
By allopurinol exposure			
Never use	9,256	1.15 (1.12 to 1.18)	1.01 (0.98 to 1.05)
Current use (<31 days)	1,864	1.31 (1.26 to 1.37)	1.05 (0.99 to 1.12)
Recent use (31–91 days)	766	1.19 (1.11 to 1.27)	1.04 (0.94 to 1.14)
Past use (>91 days ago)	1,202	0.90 (0.85 to 0.95)	0.73 (0.67 to 0.80)

Abbreviations: HR: indicates hazards ratio; CI, confidence interval; PY, person years.

(a) Adjusted for age, sex, body mass index, smoking status, alcohol use, the most recently recorded estimated glomerular filtration rate in the past year, a history of cancer, stroke and the use of an urinary catheter, systemic corticosteroids, non-insulin antidiabetic drugs and insulin 6 months before.

Table 4 shows that there was no association between infection-related mortality due to pneumonia and exposure to gout versus controls (adj. HR 1.03, 95% CI 0.93 to 1.14). Stratification by use of gout medication also did not alter the risk estimates.

**Table 4:** Risk of infection-related mortality due to pneumonia, in patients with gout compared to matched controls, stratified by use of colchicine and allopurinol.

	No. of events (death) (n = 1,965)	Age/sex adjusted HR (95% CI)	Adjusted HR^a^ (95% CI)
No Gout	1,390	Reference	Reference
Gout	575	0.97 (0.88 to 1.07)	1.03 (0.93 to 1.14)
By colchicine exposure			
Never use	475	0.99 (0.89 to 1.10)	1.07 (0.96 to 1.19)
Current use (<31 days)	<5 events^b^	0.67 (0.25 to 1.78)	0.65 (0.24 to 1.74)
Recent use (31–91 days)	<5 events^b^	0.61 (0.27 to 1.36)	0.61 (0.27 to 1.37)
Past use (>91 days ago)	92	0.92 (0.76 to 1.12)	0.92 (0.75 to 1.13)
By allopurinol exposure			
Never use	384	0.98 (0.87 to 1.10)	1.06 (0.94 to 1.19)
Current use (<31 days)	91	1.20 (0.97 to 1.48)	1.22 (0.98 to 1.52)
Recent use (31–91 days)	30	0.89 (0.63 to 1.25)	0.95 (0.67 to 1.33)
Past use (>91 days ago)	70	0.93 (0.73 to 1.18)	0.97 (0.76 to 1.23)

Abbreviations: HR: indicates hazards ratio; CI, confidence interval; PY, person years.

(a) Adjusted for age, sex, body mass index, smoking status, alcohol use, the most recently recorded estimated glomerular filtration rate in the past year, a history of dementia, dysphagia, stroke, epilepsy, ischaemic heart disease, sepsis and the use of bronchodilators, inhalation corticosteroids, systemic corticosteroids, non-insulin anti-diabetic drugs and insulin within 6 months before, the use of influenza vaccinations 1 year before and the use of pneumococcal vaccinations 5 years before.

(b) According to Independent Scientific Advisory Committee (ISAC) guidance on the content of protocols for research using CPRD data no cell containing <5 events are reported.

Table 5 shows that no association between infection-related mortality due to UTI and exposure to gout versus controls was observed (adj. HR 1.16, 95% CI 0.98 to 1.37). Stratification by use of gout medication revealed that never use of colchicine (adj. HR 1.25, 95% CI 1.04 to 1.49) and recent allopurinol use (adj. HR 1.56, 95% CI 1.00 to 2.43) were associated with an increased risk of mortality due to UTI.

**Table 5:** Risk of infection-related mortality due to UTI, in patients with gout compared to matched controls, stratified by use of colchicine and allopurinol.

	No. of events (death) (n = 697)	Age/sex adjusted HR (95% CI)	Adjusted HR^a^ (95% CI)
No Gout	454	Reference	Reference
Gout	243	1.19 (1.01 to 1.40)	1.16 (0.98 to 1.37)
By colchicine exposure			
Never use	195	1.24 (1.04 to 1.48)	1.25 (1.04 to 1.49)
Current use (<31 days)	<5 events^b^	0.94 (0.23 to 3.76)	0.75 (0.19 to 3.02)
Recent use (31–91 days)	<5 events^b^	1.48 (0.62 to 3.60)	1.23 (0.51 to 2.99)
Past use (>91 days ago)	42	1.12 (0.82 to 1.52)	0.99 (0.72 to 1.36)
By allopurinol exposure			
Never use	158	1.14 (0.95 to 1.37)	1.14 (0.94 to 1.38)
Current use (<31 days)	31	1.15 (0.79 to 1.67)	1.03 (0.71 to 1.51)
Recent use (31–91 days)	20	1.62 (1.04 to 2.52)	1.56 (1.00 to 2.43)
Past use (>91 days ago)	34	1.27 (0.89 to 1.82)	1.22 (0.85 to 1.74)

Abbreviations: HR: indicates hazards ratio; CI, confidence interval; PY, person years.

(a) Adjusted for age, sex, body mass index, smoking status, alcohol use, the most recently recorded estimated glomerular filtration rate in the past year, a history of dementia, dysphagia, stroke, epilepsy, ischaemic heart disease, sepsis and the use of bronchodilators, inhalation corticosteroids, systemic corticosteroids, non-insulin anti-diabetic drugs and insulin within 6 months before.

(b) According to Independent Scientific Advisory Committee (ISAC) guidance on the content of protocols for research using CPRD data no cell containing <5 events are reported.

## Discussion

This study showed that the risk of pneumonia, UTI or infection-related mortality was not reduced in patients with gout compared to population-based controls. Moreover, stratification by treatment with colchicine did not identify subgroups with reduced risks for pneumonia, UTI or infection-related mortality either, while stratification by treatment with allopurinol only showed a decreased risk of UTI in past users. On the contrary, we found that patients with gout had an elevated risk of pneumonia. Therefore, the results of the present study were not in line with our main hypothesis that patients with gout would have a decreased risk of acquiring infections. In addition, our findings regarding our secondary hypotheses, i.e. that treatment with colchicine (because of its immunosuppressive effects) or allopurinol (because it reduces sUA and therefore neutralizes the protective role of sUA) might enhance infections were inconsistent.

To the best of our knowledge, this is the first study that evaluated the risk of community-acquired infections in patients with gout versus matched controls. A previous population-based study by Lim *et al*.^21^, investigated the risk of septic arthritis in patients with gout, and revealed that gout patients had a 2.6-fold increased risk of developing septic arthritis when compared to non-gouty controls. However, the protective effect of a pro-inflammatory state is unlikely to play a major role in the pathophysiology of septic arthritis in these patients. Local joint damage due to gout, and possibly arthrocentesis and intra-articular injections, but also misdiagnosis of the disease, since gout and septic arthritis have similar disease features, might have contributed to this increased risk. Our study also showed increased risks of infection in certain subgroups, although only for pneumonia this risk persisted after adjusting for classic confounders. The risk of UTI disappeared after statistically adjustment for confounders (chronic kidney disease, systemic corticosteroids, use of anti-diabetics, sex and use of an urinary catheter). From a pathophysiological view, there is no good reason why gout itself should increase the risk of infections after adjusting for confounders. It is possible that a true inverse association may have not been detected due to a wide range of epidemiological limitations, such as misclassification bias, detection bias or residual confounding. Although we were able to adjust for many possible confounders, it is likely residual confounding still has occurred. First, our data show that gout patients have a higher BMI, suffer more often from chronic kidney disease, asthma and/or COPD and diabetes, and were also more likely to use systemic glucocorticoids. Despite the fact we have adjusted for these confounders in our analysis, we acknowledge we were unable to correct for all potential confounders such as under-ascertained comorbidities and for the severity of health impact of some confounders, which occur more often in gout patients. Second, another concern is that glucocorticoids, which are well-known to increase the risk of infections, were prescribed more to gout patients. Although this was adjusted for, it may have introduced information bias, while exposure to glucocorticoids was measured, as for all drugs, from prescriptions. As such, we have no information whether these drugs were actually taken. Not taking these drugs (adherence is a well-known problem in gout patients) could have led to an underestimation of the true risk. Third, missing data with respect to smoking, BMI or eGFR might have introduced residual confounding as a result of imperfect adjustment of these variables.

Although our data do not support our hypothesis of a net pro-inflammatory state that enhances resistance to infections in patients with gout, it is too early to reject the hypothesis based on these data alone. Trained immunity (or innate immune memory) is mediated by epigenetic reprogramming of monocytes and/or macrophages^22^. This reprogramming when induced by BCG is present for approximately 1 year^23^. However, it is possible that sUA-induced trained immunity is not so long lasting as was demonstrated for BCG or bèta-glucan^24^. It is also possible that the clinical protective effect of sUA is small or might be counteracted by other mechanisms (such as residual confounding, or the fact this is a population-based cohort in which sUA levels are not sharply increased).

The impact of treatment with allopurinol and colchicine was more difficult to explain due to inconsistent findings. It was interesting to see that especially past allopurinol use was associated with a decreased risk of UTI compared to controls. A possible explanation might be that the rise in sUA after discontinuation of allopurinol (non-compliance to allopurinol is well recognized^25^) is protective in acquiring infections, while in current and recent users allopurinol might neutralize the protective effect of sUA. With regard to pneumonia, never using allopurinol was associated with the lowest infection risks. These patients might have been exposed more consistent to higher sUA levels over time, but still might have an increased risk when compared to controls due to residual confounding. For colchicine we hypothesized that it enhances infections because of its anti-inflammatory and immune-modulatory effects. Case-reports and case studies have shown that patients with colchicine overdose develop infectious complications^26–28^, also in the absence of neutropenia, a well-established side effect^29^. Also, in patients using normal doses of colchicine and with normal cell counts, inhibitory effects of colchicine on leukocyte functions, such as degranulation^30^, chemotaxis and adherence^31^, might be present. However, it is of note that the use of colchicine for acute gout in the UK is limited. Moreover, the effects were not consistent with the expected direction. Finally, it cannot be excluded that patients with current colchicine use actually had an infection that triggered an acute attack/flare and therefore a prescription of colchicine.

Our hypothesis that patients with gout have a reduced risk of infection-related mortality, could not be confirmed. This finding is in line with findings from a study on mortality in patients with gout that had specified causes of death, other than cardiovascular death^32^. This retrospective cohort study that used the National Death Registry in Taiwan showed that women with gout had an excess mortality due to infectious diseases (standardized mortality ratio 2.25) compared to women without gout. However, this association disappeared after multivariate adjustment. Literature on mortality in patients with gout has shown that patients with gout have an increased risk of cardiovascular mortality, as well as all-cause mortality^33^. As such, it would be interesting to investigate whether patients with gout, who had acquired an infection might die because of cardiovascular causes rather than due to infections. Not in the least, because it is known that infections might exacerbate underlying cardiovascular disease^34^. This was however beyond the scope of this study.

Our study has several strengths. It has a large sample size, used general population data and had a follow-up. The large amount clinical information routinely and longitudinally collected in clinical practice, allowed us to statistically adjust for many potential confounders such as, age, sex, smoking status, alcohol use, comorbidity and use of medication.

Our study has other limitations that need to be addressed. First, sUA levels, which play a key role in our hypothesis, are not routinely collected in CPRD. Patients with gout therefore acted as a surrogate representing the status of hyperuricaemia. Second, the use of diagnostic codes, mainly registered by general practitioners rather than rheumatologists, to define gout might have resulted in non-differential misclassification of the outcome and a bias towards null. Still, gout, but also the outcomes (pneumonia, UTI and mortality) are already extensively studied in CPRD and studies performed with CPRD have been extensively validated^16, 35^.

Third, diagnostic bias may have masked a protective effect of gout on infections, because patients with gout may more often visit their general practitioner compared to controls. This may have increased their likelihood to be diagnosed with a pneumonia or UTI.

In conclusion, this study did not support the hypothesis that patients with gout may acquire fewer community-acquired infections, such as pneumonia and UTI or have lower infection-related mortality. In contrast, this study showed that patients with gout had an increased risk of pneumonia and UTI, although for the latter these increased risks were attributable to classic risk factors. Therefore, the clinical relevance of these findings remain unclear and the effects seem small. Future research is needed to elucidate the exact mechanisms between uric acid, interleukins, infections and the role of colchicine and allopurinol.
